# Mental health assessment of healthcare workers in the emergency department of a low middle-income country during COVID-19 pandemic

**DOI:** 10.1186/s12991-022-00426-x

**Published:** 2022-12-03

**Authors:** Shahan Waheed, Nirdosh Kumar, Bushra Qaiser Qureshi, Ahmed Rahim

**Affiliations:** Emergency Medicine, Aga Khan University Hospital Karachi, Stadium Road, PO Box 3500, Karachi, Pakistan

**Keywords:** Emergency department, Fear, Depression, Anxiety, Healthcare workers, COVID-19

## Abstract

**Introduction:**

Emergency department (ED) healthcare workers in Pakistan during the COVID-19 pandemic are facing an acute rise in mental illnesses. In this study, the authors aim to assess the frequency of anxiety and depression among healthcare workers in the ED.

**Methods:**

A cross-sectional online google form-based survey was conducted in the ED of Aga Khan University Hospital, Karachi, Pakistan between July and August 2020. The Hospital Anxiety and Depression (HAD) scale was used for mental illness assessment among ED healthcare workers. Descriptive analysis of grading as per the Likert scale is done through frequencies, means, and standard deviations. Categorical variables were expressed as frequency (%). Mann–Whitney *U* test was used to compare scores of various groups and sub-groups and the Chi-square test was used to assess the association of depression and anxiety categories among the groups.

**Results:**

In the ED, 127 healthcare workers (physicians and nurses) were included in this survey. The median depression score was 8 (IQR 6–10) with 21% (27) falling under depression and 39% (50) under borderline depression. The median anxiety score was 9 (IQR 7–12) with 33% (42) having abnormal, and 38% (48) having borderline anxiety. Healthcare workers working for > 45 h per week have odds of 3.62 [1.374–9.549] of developing depression compared to anxiety with a *p*-value of 0.009. Similarly, nurses and medical officers develop depression with odds of 2.18 [1.016–4.686] *p*-value 0.045 and 5.18 [0.197–1.02] *p*-value 0.002, respectively.

**Conclusion:**

ED healthcare workers during the COVID-19 pandemic suffered high levels of anxiety and depression, which is a matter of concern. Comprehensive support and training of ED healthcare workers are needed to promote physical and mental well-being and to develop guidelines that should be used during situations that can affect the mental health of healthcare workers.

## Introduction

Anxiety and depression are commonly reported mental illness disorders worldwide, 1 in every 8 people in the world lives with a mental disorder [1, 2]. Epidemics pose an increased demand for healthcare workers and the burden of mental illnesses is profound among healthcare workers and the emergency departments, and in the current COVID-19 pandemic, emergency department healthcare workers are sharing a major burden worldwide [3, 4]. A load of mental illness is plagued by variability in reported prevalence rates and a lack of published data from low-resource settings like Pakistan [5]. Previous studies from Pakistan do report an overall prevalence of anxiety and depression in the community to be 34% (range 29–66% among women and 10–33% for men) [1, 5, 6]. ED healthcare workers by their job are at increased risk of mental health problems [7]. The emergence of COVID-19 in Pakistan developed an unprecedented situation for ED healthcare workers at the front-line and making decisions under extreme pressure. The decisions made are influenced by the factors, like scant resources, the practice of justice and equity, and keeping in deliberation one’s physical and psychological needs responsibly.

The assessment of mental illness in the ED includes anxiety and depression, which are not routinely assessed. The studies report that developing countries have almost two-thirds of psychiatric patients in the world [8, 9]. The ongoing COVID-19 pandemic with its associated stress and the weak healthcare systems may worsen the psychological impact on the medical system’s first line of defense. Healthcare workers often overlook mental illness issues during a pandemic, putting them at risk of developing anxiety and depression and, hence, poor outcomes. The previously published studies have focused broadly on mental health issues and utilized different scales [8]. Our study focuses exclusively on ED healthcare workers by utilizing the HAD scale, a validated scale in our population [10].

Therefore, this study aims to determine the frequency of anxiety and depression among healthcare workers working in ED during the COVID-19 pandemic in Pakistan and explore the personal, social, and environmental factors that were responsible for workplace anxiety and depression.

## Methods

**Fig. 1:**
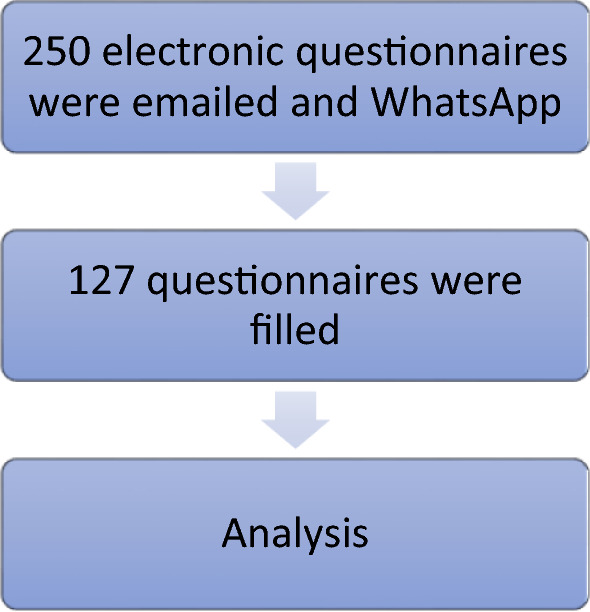
The study questionnaire was pre-tested and then circulated to the ED healthcare workers through email and WhatsApp

A cross-sectional online hospital-based survey was conducted in the ED of Aga Khan University Hospital (AKUH) between July and August 2020. The ED of AKUH is a 62-bedded facility located in Karachi, Pakistan, receiving 60,000 patients annually. The study participants were selected through consecutive sampling and included ED physicians and nurses involved in patient care during the COVID-19 pandemic. Participants with known psychiatric disorders or ailments related to stress, like psoriasis and irritable bowel syndrome, were excluded. The reason for excluding these two groups is that these disorders have the potential of causing mental illness and may not represent the true effect of COVID-19 on healthcare staff. Moreover, as we did not have previous baseline data on mental illness among ED healthcare workers, we aim to exclude all factors that may falsely estimate the current burden of mental health illness among ED healthcare workers. The study questionnaire was pre-tested and then circulated to the ED healthcare workers through email and WhatsApp Fig. 1.

The questionnaire consists of demographic variables, like age, sex, and components of the Hospital Anxiety and Depression (HAD) scale. It contains seven questions for anxiety and seven questions for depression. The HAD scale was chosen because of its validation in previous studies on our population. It is one of the National Institute for Health and Care Excellence (NICE) recommended tools for the diagnosis of depression and anxiety among healthcare workers. Additionally, data on associated factors, like the number of duties per week, number of hours per week, availability of personal protective equipment (PPE), and number of hours working with PPE per week, were also collected. The cut-off scores for quantification were taken as previously tried and tested in the literature. The primary outcome was the frequency of anxiety and depression among healthcare workers in the ED.

### Statistical analysis

The analysis was based on respondents who provided complete data. Descriptive statistics are presented as frequencies and percentages for categorical variables. Basic descriptive statistical analysis of grading as per the Likert scale is done by calculating frequencies, mean, and standard deviations. Normality of the distribution of age (years), depression, and anxiety scores were measured by applying the Shapiro–Wilk test and a *p*-value < 0.05 is considered statistically significant. Median and interquartile range (IQR) were computed, and the Mann–Whitney *U* test was used to compare scores by various groups and sub-groups of participants. Categorical variables were expressed as frequency (%) and the Chi-square test was applied to assess the association of depression and anxiety, among various groups and sub-groups of participants. The percentage of grading of all ED healthcare workers was calculated by combining the frequency of each item in the questionnaire. The responses on the Likert scale were totaled for each item. The data were entered and analyzed using IBM SPSS statistical package for windows version 20.

## Results

A total of 127 participants were included in this survey with a response rate of 51%. Hospital Anxiety and Depression Scale (HADS) for all two domains with Cronbach's alpha coefficient was 0.85 [95% CI 0.808–0.886], inter-rater reliability of depression score was 0.722 [95% CI 0.64–0.791], and anxiety was 0.779 [95% CI 0.713–0.833]. The median depression score was 8 [IQR: 6–10] with 21% (27) falling under the abnormal depression category and 39% (50) with normal to borderline depression. The median anxiety score was 9 [IQR: 7–12] with 33% (42) having abnormal and 29% (37) normal and 38% (48) with borderline anxiety. 35% (45) of them were Registered Nurses, 32% (40) were medical officers, and only 8% (10) were a consultant. Most participants were under the age of 30 years 63% (80) and almost 78% (99) had working ED experience of fewer than five years. 70% (89) of participants found spent more than 45 h in the ED and were directly or indirectly exposed to COVID-19 in suspected and confirmed patients. The demographic profile, year of experience, and COVID-19 exposure of participants are presented in Table 1.

**Table 1:** Demographic profile, year of experience, and COVID-19 exposure of the participants

Characteristics	Total
Total (*N*)	127
*Median [IQR]*	
Depression score	8 [IQR: 6–10]
Anxiety score	9 [IQR: 7–12]
*Cronbach's alpha coefficient*	
Overall reliability	0.85 [95% CI 0.808–0.886]
Depression	0.722 [95% CI 0.64–0.791]
Anxiety	0.779 [95% CI 0.71–0.833]
*Designation*	
Resident	25% (32)
Medical Officer	32% (40)
Registered Nurse	35% (45)
Consultant	8% (10)
*Age groups*	
≤30 Years	63% (80)
>30 Years	37% (47)
*Years of experience working in the ED?*	
≤5 Years	78% (99)
>5 Years	22% (28)
*How many hours do you spend working in the ED per week?*	
≤45 Hours	30% (38)
>45 Hours	70% (89)
*Anxiety*	
Normal (0–7)	29% (37)
Borderline (8–10)	38% (48)
Abnormal (11–21)	33% (42)
*Depression*	
Normal (0–7)	39% (50)
Borderline (8–10)	39% (50)
Abnormal (11–21)	21% (27)
*Consent scientific purpose*	
Yes	98.4% (125)
No	1.6% (2)

Similarly, categories of anxiety and depression were found to be comparatively higher among participants who had an age group of fewer than 30 years (*p*-value = 0.007*), and most of the registered nurses found significantly abnormal depression (*p*-value = 0.015*). Participants who have abnormal depression and anxiety were found to spend more hours in an emergency (*p*-value = 0.016*). Depression and anxiety levels stratified by the COVID-19 status of the participant are presented in Table 2.

**Table 2:** Depression and anxiety levels stratified by the COVID-19 status of the participant

	Anxiety				Depression			
	Normal	Borderline	Abnormal	*P*-value	Normal	Borderline	Abnormal	*P*-value
*N*	37	48	52		50	50	27	
*Age groups*								
≤ 30 Years	14.2% (18)	21.3% (27)	26.8% (34)	0.007*	20.5% (26)	26% (33)	15.7% (20)	0.126
>30 Years	15% (19)	16.5% (21)	6.3% (8)		18.9% (24)	13.4% (17)	5.5% (7)	
*Designation*								
Resident	3.9% (5)	9.4% (12)	11.8% (15)	0.015*	6.3% (8)	9.4% (12)	9.4% (12)	0.048*
Medical Officer	12.6% (16)	15% (19)	3.9% (5)		15% (19)	11% (14)	5.5% (7)	
Registered Nurse	10.2% (13)	9.4% (12)	15.7% (20)		15.7% (20)	13.4% (17)	6.3% (8)	
Consultant	2.4% (3)	3.9% (5)	1.6% (2)		2.4% (3)	5.5% (7)	0% (0)	
*Hours spending ED*								
≤45 Hours	12.6% (16)	12.6% (16)	4.7% (6)	0.016*	12.6% (16)	12.6% (16)	4.7% (6)	0.616
>45 Hours	16.5% (21)	25.2% (32)	28.3% (36)		26.8% (34)	26.8% (34)	16.5% (21)	
*Working experience ED*								
≤5 Years	21.3% (27)	28.3% (36)	28.3% (36)	0.325	30.7% (39)	29.1% (37)	18.1% (23)	0.528
> 5 Years	7.9% (10)	9.4% (12)	4.7% (6)		8.7% (11)	10.2% (13)	3.1% (4)	

The odds of participants screened positive for severe depression and anxiety with age, year of service in the ED, working hours in the past 7 days, year of residency, nature of the job, and personal protective equipment worn for how many hours are presented in Table 3.

**Table 3:** Odds of participants being screened positive for severe depression, and anxiety, with demographic characteristics and pre-existing study characteristics

Study characteristics	Depression		Anxiety	
	OR [95% CI]	*P*-value	OR [95% CI]	*P*-value
Age groups	3.78 [1.567–9.109]	0.003*	0.63 [0.308–1.267]	0.192
Working Experience	0.48 [0.177–1.286]	0.144	0.55 [0.173–1.751]	0.312
Hours Spending	3.62 [1.374–9.549]	0.009*	1.65 [0.606–4.477]	0.328
Consultant	0.48 [0.098–2.374]	0.369		–
Registered Nurse	2.18 [1.016–4.686]	0.045*	1.4 [0.556–3.502]	0.479
Medical Officer	5.18 [0.197–1.027]	0.002*	0.71 [0.273–1.849]	0.484
Resident	0.45 [1.851–1.027]	0.058	3.2 [1.296–7.898]	0.012*

Anxiety score was significantly higher among healthcare workers, i.e., residents who were directly or indirectly exposed to COVID-19 suspected or confirmed patients at work (odd ratio 3.2 [95% CI 1.296–7.898]; *p* = 0.012*). However, the odds of participants who had Working Experience and spent more hours in the ED were not associated with depression and anxiety. Participants who have aged less than 30 years had 3.7 higher anxiety [odd ratio 3.78 [1.567–9.109], *p* = 0.003*], additionally, nurses and medical officers had higher depression as compared to residents and consultants [odd ratio 2.18 [1.016–4.686], *p* = 0.045*, vs. 5.18 [0.197–1.027], *p* = 0.002*], respectively.

**Fig. 2:**
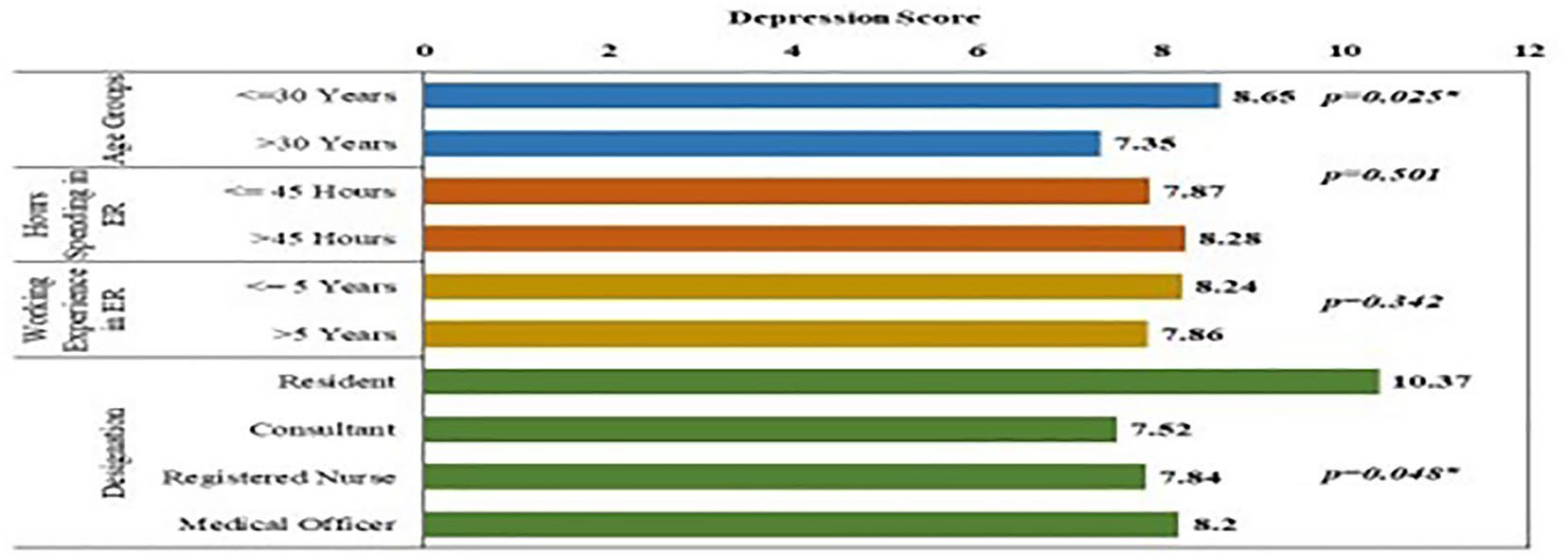
Bar chart showing depression scores among different groups of study participants

**Fig. 3:**
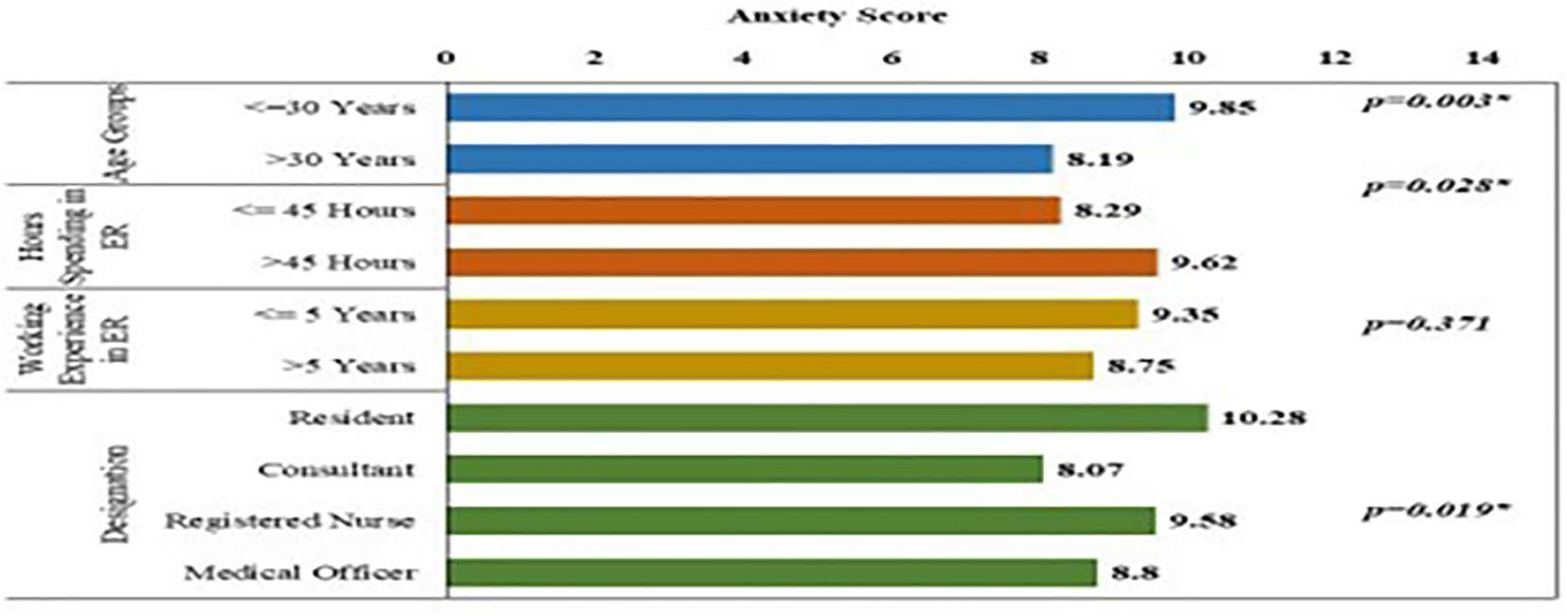
Bar chart showing anxiety scores among different groups of study participants

Depression and anxiety scores among different participant sub-groups are shown in Figs. 2 and 3.

## Discussion

Our study highlights the increased incidence of mental health illness among ED healthcare workers during the COVID-19 pandemic. The reasons for this are manifold. A few that are worth mentioning are the risk of contracting the disease from the patients and the fear of transmitting the disease to their own families [11]. The ED being the front-line service in dealing with COVID-19 patients was a privileged observatory of the general population with COVID-19-related fears. It admitted patients with or without psychiatric diagnoses, fearing COVID-19 disease, or the related sanitary measures and this cohort included the ED healthcare workers, their relatives, or colleagues. The results of this article also propose the refinement of individualized and long-term sanitary measures strategies with a focus on an oriented prevention policies strategy [12].

The factors that are highlighted in our paper are important as it paves the way to develop mental health assessment and training programs for healthcare workers working in high-risk environments, like ED and ICU.

The study is important from Pakistan, as it is the only study to date that has focused on the mental health assessment of ED healthcare workers during the COVID-19 pandemic. Moreover, the factors that are explored can later be intervened to either minimize or decommission the risk of mental health illness among healthcare workers in the ED.

Although some published literature has looked at healthcare workers’ assessment of mental health during the COVID-19 pandemic, a few have focused on ED healthcare workers (physicians and nurses) [13].

Our study reports a significant involvement of mental health disorders among medical officers and nurses. This is congruent with the previous research demonstrating widespread negative attitudes among healthcare workers in the ED [1, 13]. The involvement of medical officers and nurses seems to be directly related to the increased number of working hours. The presence of these psychological disorders among front-line healthcare workers suggests that they must cope with psychological distress and are at risk of allostatic overload. The increased frequency of depression and anxiety among those who are less than 30 years of age might be due to their less ED working experience and coping mechanisms that are not well-developed to deal with the stress of ED. The COVID-19 pandemic has come up with an added burden to the ED healthcare workers due to added stress, insomnia, the burden of wearing personal protective equipment for long hours, fear of getting infected and subsequently putting their families at risk and patient’s ignorance of not accepting that reality, and seriousness of COVID 19 infection [14].

COVID-19 has affected many front-line healthcare workers. In low-resource settings like Pakistan, it increased the development of psychological problems among healthcare workers who are undoubtedly at increased risk of contracting the disease. The emergency departments of the city are currently working at their full capacity. The increased workload and the stressors of long duty hours may worsen the situation by delving into mental health illnesses. The lack of structured programs and training addressing this important issue is not existent. Additionally, the lack of standard operating procedures, lack of resources, and prolonged wearing of personal protective equipment might make the situation further gloomy. Pandemics bring significant economic crises along with medical, psycho-social, and physical health crises, and in times of economic crises, the only statistically significant protective factor in mitigating mental health effects is the “interpersonal trust” as observed during the 2008–2009 global financial crises [15, 16]. However, the future challenge is probably not interpersonal trust only, but the improvement of trust in institutions, government, policymakers, and the information of rights as well [17–19].

Our study findings report an impact on healthcare workers' mental health as reported in China and Pakistan [5, 8, 20]. The frequency of depression and anxiety was in both nurses and physicians, but nurses were in the majority. The reason could be due to their long working hours and insufficient skills for coping and resiliency.

Despite these implications stated above, the study had a few limitations. First, it included healthcare workers from a single center and the results may not be generalizable to other settings. However, the frequency of depression and anxiety might help in the inference of the situation of the other healthcare setups in the city. Second, this study was cross-sectional; thus, causal conclusions should be drawn carefully. Further studies with longitudinal designs are needed to investigate the possible causal relationships and the long-term impact of depression and anxiety on ED healthcare workers. Third, all data collected were self-reported by the participants, and more objective data could be used in the future for similar research. There is a need for an exploratory design to comprehensively assess the problem. Lastly, the sample size of the study is small but its focus on the ED might make its utility important for formulating guidelines for prevention and coping skills for the front-liners who are involved in delivering care during the pandemic.

## Conclusions

In conclusion, our study reports a higher percentage of mental illnesses among ED healthcare workers. This addresses the need to have a comprehensive mental health assessment and training program for healthcare workers working in high-stress areas like the emergency department and ICU. Additionally, there is a need to remove the factors that may act as a catalyst in increasing the incidence of mental health disorders like working hours, provision of adequate rest, and the development of well-being programs aimed at empowering the psychological well-being and resilience of workers.

## Data Availability

The datasets generated and analyzed during the current study are not publicly available to maintain the confidentiality of the participants but are available from the corresponding author at reasonable request.

## Declarations

### Statement of similarity

The manuscript has been checked with dedicated software for similarity and it does not exceed the standard values for the journal. The initial similarities were due to a preprint available on the research square which is my own [21].
